# 2134: Integrating Epidemiology and Biostatistics teaching using the case method

**DOI:** 10.1017/cts.2017.160

**Published:** 2018-05-10

**Authors:** Jessica K. Paulus, Angie Rodday, Farzad Noubary

## Abstract

OBJECTIVES/SPECIFIC AIMS: Biostatistics and Epidemiology courses within clinical research or public health training programs are typically developed and taught separately. As a result, students may have trouble in their research outside the classroom, where biostatistical and epidemiological concepts must be well integrated. Case method teaching is a participant- and discussion-centered pedagogical approach that has been used in business and law schools for more than 50 years to improve student learning, yet has taken longer to be adopted in health professional schools. The case method is distinguished by presenting learners with a real-world problem without a single unique solution. Designed to mimic the constraints and incomplete information found in real life, it is an ideal approach for integrating multiple related disciplines. A team of Clinical and Translational Science (CTS) faculty from the Tufts CTSI collaborated to develop a new course that integrates epidemiology and biostatistics disciplines using the case method. METHODS/STUDY POPULATION: We developed an intermediate-level, case-based course integrating epidemiology and biostatistics topics using modern, real-world clinical examples. Recognizing the importance of technical skill building, this intermediate-level Tufts CTS course adopted a hybrid approach, incorporating lecture and in-class laboratory exercises, alongside cases. We surveyed CTS faculty to identify a set of core methodological competencies. These included randomized trials, case-control and cohort studies, confounding, effect modification, propensity scores, linear and logistic regression, and survival analysis. Faculty provided us with clinical questions and deidentified data sets corresponding to these competencies; we also reviewed publicly available data sets. RESULTS/ANTICIPATED RESULTS: CTS faculty collaborated to develop 10 cases (with accompanying data sets) from modern clinical research examples that illustrate the connections between epidemiology and biostatistical concepts. Each case contains a background section, a statement of the core problem, a data set with data dictionary, articles from the primary literature (often the publication of the data set) with discussion questions and in-class lab exercises (R programming). One case presents students with the challenge of whether acupuncture may be an effective therapy for pain associated with chronic headache. Through case activities, students gain experience weighing observational Versus experimental evidence, apply directed acyclic graph theory, and analyze clinical trial data. Qualitative evaluations in 2015 (pilot year) and 2016 indicate students preferred the integrated approach to separate courses, and found the integration facilitated application of methods to their independent research projects. Significant rewards for faculty include cross-disciplinary collaboration, sharpened teaching skills, and engaging with learners in a dynamic classroom environment. DISCUSSION/SIGNIFICANCE OF IMPACT: Despite administrative and pedagogical challenges, a case-based, integrated curriculum offers rewards for faculty and students. The case method may be a useful pedagogical strategy to integrate other closely related topics or courses in translational science to better prepare scholars for the challenges of independent research.

